# The Behaviors and Habits of Young Drivers Living in Small Urban Cities

**DOI:** 10.3390/ijerph22020165

**Published:** 2025-01-26

**Authors:** Alexander M. Crizzle, Mackenzie L. McKeown, Ryan Toxopeus

**Affiliations:** School of Public Health, University of Saskatchewan, Saskatoon, SK S7N 2Z4, Canada; mackenzie.mckeown@usask.ca (M.L.M.); rtoxopeu@gmail.com (R.T.)

**Keywords:** young driver, driver behaviors, alcohol consumption, distracted driving, driver training

## Abstract

While studies have typically examined the driving habits of young drivers living in large urban cities, few have examined the habits of young drivers living in smaller cities with large rural surrounding areas. Three surveys were disseminated to 193 young drivers, 65 police officers, and 62 driving instructors to examine the driving habits and challenging driving situations young drivers experience. Almost a fifth (18.1%) reported consuming alcohol prior to driving; alcohol consumption prior to driving was significantly associated with eating food/drinking beverages while driving, cellphone use, and speeding. The most challenging situations young drivers reported were night driving, encountering wild animals on the road, and driving in extreme weather conditions (e.g., ice, snow). Driving instructors reported that young drivers had challenges with lane positioning, speed control, and navigating traffic signs and signals. Additionally, police officers reported issuing tickets to young drivers primarily for failure to stop, distracted driving, impaired driving, and speeding. Young drivers living in smaller cities and rural communities have unique challenges, including interactions with wildlife, driving on gravel roads, and driving in poor weather and road conditions (e.g., ice, snow). Opportunities for young drivers to be exposed to these scenarios during driver training are critical for increasing awareness of these conditions and reducing crash risk.

## 1. Introduction

Young drivers, who are between the ages of 16–24 years, comprise 11.4% of all licensed drivers in Canada [1,2], yet they account for 18.6% of all injurious motor vehicle crashes and 14.4% of all motor-vehicle fatalities [2]. In developed countries such as Canada, Australia, and the USA, the high crash rates of young drivers occur due to a variety of factors, including speeding, distracted driving, and driving while under the influence of alcohol and cannabis, all of which significantly increase crash risk and endanger all road users [2,3,4,5,6,7,8,9]. Other studies in Australia, New Zealand, and Columbia, despite cultural and geographic differences, show that young drivers engage in similar risky driving behaviors, including cell phone usage, impaired driving, and driving with passengers, all of which are associated with road crashes [10]. Studies from Canada, Italy, and Australia report that between 25% and 65% of young drivers drive above the posted speed limit (i.e., speeding) [9,10,11,12,13], and young drivers who speed 10 km/h or more above the posted speed limit have a 2.77 times increased risk of being involved in a collision compared to the same group of young drivers when not speeding [11,13]. Despite the consistent findings across these studies in urban contexts, only a few studies have examined this issue in young drivers that live in small cities and rural communities. While the driving skills and abilities are not necessarily different between urban and rural drivers, the environment (e.g., weather conditions, road design) and contextual factors (distance from home, perceptions of risk-taking behaviors) are.

A recent US study found that speeding and driving under the influence were the leading contributing factors in rural area crashes, while aggressive driving was more pronounced in urban areas [14]. In Canada, almost one quarter of all fatal crashes (24.7%), regardless of age, are due to speeding [2]. In rural areas, studies show that speeding is negatively associated with distracted driving behaviors (e.g., texting while driving) regardless of age [15,16], and that texting while driving is positively associated with driving while under the influence of alcohol and/or cannabis in young drivers [14,17]. Studies report that between 8.6 and 33.3% of young drivers report using cannabis 1–6 h before driving, and that young drivers who drive under the influence of cannabis have a four times greater crash risk due to their poorer reaction time, lane position, and impaired judgment compared to young drivers not under the influence of cannabis [1,3,5,9,18,19,20,21,22]. Similarly, studies show that between 4.7 and 18.9% of young drivers report driving while under the influence of alcohol [5,9,11,22,23], which increases their risk of a crash by three times [7], through negatively altering their ability to maintain lane position and speed regulation [21,24]. However, it is likely that young drivers under-report the extent to which they engage in impaired driving due to the fear of license suspension, fines, and/or demerit points, similar to distracted driving studies.

Numerous distracted driving behaviors are associated with crash risk resulting from an inability to detect roadway changes and/or respond quickly to hazards [25]. Studies show that between 16 and 36% of young drivers commonly engage in distracted driving behaviors, such as texting while driving, which significantly increases crash risk [8,17]. In fact, some studies show that almost a quarter of fatal crashes in young drivers (22%) are a result of texting while driving [26]. In addition to texting while driving, approximately 64% and 90% of young drivers report that they read a text message/email or check social media while driving [17,27,28], which also increases crash risk by 2–20% [11,27,28].

Driving instructors are responsible for administering training programs for young drivers. Interviews with driving instructors in Australia reported that young drivers (ages 16–24) overestimated their driving ability and consequently were more likely to engage in risky driving practices (e.g., speeding, tailgating, etc.) [29]. A survey of Australian driving instructors reported that young drivers (ages 16–24) were not adequately trained on essential driving skills (e.g., emergency braking, passing a cyclist on the road) during training [30]. Despite the role and importance of driving instructors in training young drivers, there have been no Canadian studies that have captured data from driving instructors on driving situations young drivers find challenging. Similarly, while police reports are used for research studies, no studies have captured data from the police officers themselves, who are responsible for issuing citations to drivers who perform dangerous driving behaviors.

To date, there have been no research studies examining young driver crash rates and the reasons they occur in Saskatchewan. The province of Saskatchewan (SK) has approximately 1.1 million residents [31], with roughly 40% of the population living in rural areas [32]. As a result of their rural location and lack of public transportation within SK, a large portion of young drivers are operating their own vehicles for travel [33]. In Saskatchewan, young drivers (ages 16–24) comprise 6.5% of all licensed drivers; however, they have the highest prevalence of alcohol-impaired driving in the country (18.5%), greatly exceeding the national average (11.4%) [23]. Additionally, young drivers in SK face unique challenges such as extreme weather (e.g., snow, ice), roads (e.g., gravel), and encountering wildlife (e.g., deer, moose, porcupine, etc.) while driving [34]. Additionally, the infrastructure and environment of Saskatchewan vastly differ from the urban locations where most studies have examined the driving behaviors of young drivers. For example, rural roadways are generally in poorer condition (e.g., potholes, wash boards, etc.), they lack safety barriers, and they have small (or no) shoulders with steep ditches, which can result in head-on crashes and/or roll-overs on shoulders when performing evasive actions to objects/wildlife/pedestrians on/near the road.

There are limited data on the driving behaviors and practices of young drivers living in smaller cities and rural areas, which may be vastly different than prior studies that have examined the crash risk of young drivers in large urban Canadian cities [3,12] and other cities in the world [4,5,6,7,8,9,10,11,12,17,19,20,21,22]. The objectives of this study are to: (1) examine challenging driving situations (e.g., poor road conditions, merging, etc.) young drivers encounter; (2) examine problematic driving behaviors (e.g., distracted behaviors, alcohol- and cannabis-use) in young drivers; and (3) determine challenging driving situations (e.g., poor road conditions, merging, etc.) that young drivers experience from the perspective of key stakeholder groups (young drivers, police officers, and driving instructors). Findings from this study can inform the materials and training presented as part of graduated licensing programs to reduce the risk of crashes.

## 2. Materials and Methods

### 2.1. Protocol

The study was approved by the Research Ethics Board at the University of Saskatchewan [REB #2511]. Data were collected from June to October 2021 using three different online surveys for each group of participants (i.e., one for young drivers, one for police officers, and one for driving instructors) that took approximately 10 min to complete. All three surveys were hosted by Qualtrics through the University’s Canadian Hub for Applied Health Research. Each participant provided written informed consent prior to study participation.

### 2.2. Surveys

The three surveys, one for each group of participants (young drivers, police officers, and driving instructors), included two sections: The first section collected demographic information (e.g., years living/working in Saskatchewan, age, gender, location, highest level of education completed). The second section asked questions specific to the various groups of interests. For example, young drivers were asked about specific driving habits (e.g., speeding, using cell phones, driving with pets, and whether the pets sat in the front or back seat), whether they had received any citations or been involved in crashes, the use of alcohol or drugs prior to driving in the previous 6 months, and challenging driving situations they have experienced (e.g., merging or changing lanes, driving at night, extreme weather conditions). The second section of the survey for police officers included questions such as the estimated number of tickets given to drivers between the ages of 15–25 years in the previous month and, of those tickets, the location and how many were given out for each type of infraction (e.g., speeding, impaired driving, distracted driving). Additionally, police officers were asked to identify common contributing factors for crashes involving young drivers (e.g., speeding, poor weather conditions, etc.) and additional training young drivers should receive before taking their on-road test. Driving instructors were asked what situations young drivers struggle with when learning how to drive (e.g., using technology, speed control, weather conditions) and what are the most common reasons young drivers fail their on-road test (e.g., not stopping fully, left-hand turns at intersections, not checking mirrors, parallel parking). Stakeholders from all three groups pilot tested their respective surveys prior to broader dissemination to ensure the clarity and wording of questions, as well as the relevance and validity (collecting the right information).

### 2.3. Recruitment

Young drivers, defined as those between the ages of 18 and 24 years, were recruited through an online bulletin board hosted by the University, flyers posted on library bulletin boards across Saskatoon, and social media posts on Twitter, LinkedIn, and Facebook. Emails were sent by the Saskatchewan Government Insurance (SGI) to recruit police officers and driving instructors and assessors through their internal networks.

### 2.4. Study Inclusion Criteria

Young drivers were included if they were between the ages of 18–24 years, had completed the Graduated Licensing Program (GDL), and possessed a Class 5 driver’s license. The GDL is a program that educates and exposes young drivers to increasing levels of risk over various stages. The first stage is obtaining a learner’s permit at age 16 (or age 15 if registered in a high school driving program), which involves 30 h of in-class training and six hours of in-car training. Subsequently, they are required to pass a written test as well as a road test. Once young drivers complete these requirements, they will obtain a Class 5 driver’s license. The Class 5 driver’s license has two stages. The first stage (Class 5 Novice 1) is to practice driving for 6 months with restrictions (e.g., only one non-family passenger allowed). After 6 months, the driver can complete and pass a second road test, which grants the driver a Class 5 Novice 2 driver’s license with certain restrictions (e.g., cannot have more passengers than seat belts available). After 12 months of driving incident-free, drivers are then granted their full license (with no restrictions).

Police officers were included if they had been a police officer for at least two years and the majority of their duties involved traffic enforcement.

Driving instructors/assessors were included if they were registered as a driving instructor with SGI, had been a driving instructor/assessor for at least two years, and worked with young drivers between the ages of 15–18.

### 2.5. Data Analysis

Continuous variables were summarized using means (M), standard deviations (SD), and ranges (M ± SD, range), and categorical variables were summarized using frequencies (n/f) and percentages (%). Chi-squared analyses were conducted to examine the associations between categorical variables. Significance was established at *p* < 0.05, two-tailed. All analyses were performed using the IBM Statistical Package for the Social Sciences (SPSS) Version 28.

## 3. Results

### 3.1. Young Drivers

Surveys were completed by 193 young drivers: mean age of 20.6 ± 1.8 years (range 18 to 24). Over half of the young drivers were female (57.0%), with almost three quarters living in urban centers (76.2%). Most respondents had their full license (88.1%); 11.9% had a novice license permit. Less than half (40.9%) drove for at least 5 years; 65.3% reported driving 5 or more days per week. Within the past 6 months, 8.1% of young drivers had received a traffic violation citation, and 6.7% of drivers had been involved in at least one crash; over one-third (38.5%) were at-fault.

### 3.2. Alcohol and Illicit Drug Consumption Prior to Driving

Almost a fifth (18.1%) reported consuming alcohol prior to driving; of those, 68.6% said they had one drink, 28.6% had 2–3 drinks, and 2.9% had 4–5 drinks. About 22% of the rural young drivers reported drinking prior to driving compared to 17% of urban drivers; differences were not significant. Approximately 6.2% reported using cannabis, and 1.6% reported consuming a cannabis-based edible before driving. Of those who consumed cannabis before driving, 15.4% said they had consumed cannabis less than an hour before driving. Approximately 69.2% of young drivers who used cannabis before driving claimed to feel safe when they drove. Only a small proportion (2.1%) used illicit drugs (e.g., cocaine, methamphetamine, MDMA) before driving.

As shown in Table 1, alcohol consumption prior to driving was significantly associated with eating food/drinking beverages while driving (χ^2^ = 6.89, *p* = 0.032), cellphone use (χ^2^ = 11.7, *p* = 0.003), and speeding (χ^2^ = 9.58, *p* = 0.008).

### 3.3. Distracted Driving Behaviors

Table 2 shows the distracted driving behaviors reported by young drivers. More than half (57.5%) used their cell phone while driving to change songs/play music (49.7%), program/adjust/view GPS (37.8%), make hands-free calls (57.0%), check notifications (22.8%), check the time (21.2%), type and send texts (35.0%), receive calls not using any hands-free device (12.4%), check social media (8.3%), take photos (6.7%), search the internet (4.1%), and read emails (1.0%). About 35.2% of young drivers drove with pets in their vehicle; 50% placed their pet in the front passenger seat, and less than half (41.2%) of respondents put a seatbelt on their pet.

### 3.4. Challenging Driving Situations

Young drivers reported having problems with a variety of driving situations, as shown in Table 3. The most challenging situations young drivers reported were night driving, followed by merging/changing lanes, bright/sunny conditions, and passing other vehicles. Almost all participants wear a seatbelt, both as the driver and passenger. Additionally, almost a quarter of young drivers reported difficulty driving on gravel roads (20.7%). No significant differences emerged with the exception that young male drivers were significantly more likely to have difficulties merging/passing other vehicles compared to young female drivers (χ^2^ = 7.33, *p* = 0.007).

Most young drivers (82.9%) encountered wild animals on the road while driving; 70.0% needed to take evasive action to avoid a collision. Almost two-thirds (65.8%) reported difficulty driving in extreme weather conditions such as ice/slippery roads (58.5%), snow (35.8%), heavy rain (26.9%), fog (16.6%), and high winds (9.3%). About 76.2% of young drivers reported driving while fatigued, with 6.8% having fallen asleep at the wheel.

Young drivers who had problems merging or changing lanes were significantly more likely to have received a citation (χ^2^ = 6.01, *p* = 0.014). Additionally, young drivers who used their cell phones while driving were significantly more likely to have received a citation (χ^2^ = 4.87, *p* = 0.027). Since there were so few crashes reported by young drivers within this sample (6.7%), crashes were not significantly associated with any distraction/procedure collected in the survey.

### 3.5. Police Officers

Police officers were aged 42.1 ± 7.9 years (range 26 to 58). About 95% of police officers were from large metropolitan cities, with the remaining 5% being from smaller communities. As shown in Table 4, at intersections, police officers had given tickets for driver errors such as failure to stop (83.1%) and distracted driving (60.0%). On main streets, police officers ticketed young drivers for impaired driving (66.2%), distracted driving (63.1%), and improper signaling (50.8%). Relatively few citations were reported on residential streets; however, more than a quarter of police officers ticketed young drivers in residential areas for impaired driving (27.7%) and a failure to stop (27.7%). There were few citations on side roads, with less than a fifth of officers ticketing young drivers for speeding (18.5%), impaired driving (18.5%), and failure to stop (18.5%). On highways, police officers most often ticketed young drivers for speeding (70.8%).

Police officers reported the most common reasons for providing a distracted driving citation were holding, viewing, or manipulating a hand-held cellphone while driving (96.9%), followed by reading (86.2%), smoking (75.4%), playing loud music (75.4%), personal grooming (72.3%), adjusting the radio or CD player (69.2%), or GPS (66.2%), and eating or drinking (58.5%).

They also reported the most common reasons young drivers are involved in crashes were related to distracted driving (87.7%), followed by driving too fast for conditions (84.6%), driver inexperience/confusion (80.0%), following vehicles too closely (61.5%), driving on poor road condition (surface or structure) (47.7%), driving in poor weather conditions (41.5%), failure to yield (38.5%), and evading animals (6.2%).

Police officers reported that young drivers should receive training in driving in snow (76.9%), followed by driving on slippery roads (69.2%), speed control (55.4%), merging with traffic (52.3%), checking blind spots (49.2%), identifying potentially dangerous situations (47.7%), signaling (44.6%), driving in rain (41.5%), and navigating heavy traffic (40.0%).

### 3.6. Driving Instructors

Surveys were completed by 62 driving instructors, who were an average age of 53.3 ± 10.4 years (range 31 to 70). About 58.1% of the driving instructors were from cities, while 41.9% were from rural locations. Driving instructors had worked on average 15.8 ± 10.9 years (range 2 to 50).

Driving instructors reported the most challenging situations for young drivers, when learning to drive, were related to lane positioning (72.6%), checking blind spots (69.4%), identifying potentially dangerous situations (61.3%), speed control (61.3%), navigating heavy traffic (61.3%), merging with traffic (61.3%), driving in slippery road conditions (59.7%), navigating traffic signs and signals (58.1%), navigating pedestrians (50.0%), and driving in snow (48.4%).

Almost a fifth (19.4%) of the driving instructors also performed the on-road driving examination for young drivers as part of the graduated licensing program. The most common reasons young drivers failed the on-road tests were not stopping completely at a stop sign or traffic light (58.3%), parallel parking (33.3%), difficulty driving in traffic (33.3%), left-hand turns at intersections (25.0%), improper use of traffic signals (25.0%), and not checking their mirrors regularly (25.0%).

### 3.7. Opportunities for Enhancing Training for Young Drivers

As shown in Table 5, police officers were significantly more likely to report that young drivers needed training on using cruise control (χ^2^ = 7.51, *p* = 0.006), driving at night (χ^2^ = 6.72, *p* = 0.010), in the rain (χ^2^ = 6.22, *p* = 0.013), and snow (χ^2^ = 11.1, *p* < 0.001) compared to driving instructors. Conversely, driving instructors were significantly more likely to report that young drivers need additional training on shifting gears (χ^2^ = 5.11, *p* = 0.024), pedal use (χ^2^ = 4.58, *p* = 0.32), lane positioning (χ^2^ = 22.2, *p* < 0.001), checking blind spots (χ^2^ = 5.32, *p* = 0.021), driving in heavy traffic (χ^2^ = 5.75, *p* = 0.016), using signals (χ^2^ = 14.7, *p* < 0.001), and watching out for pedestrians and cyclists (χ^2^ = 12.6, *p* < 0.001). While not significantly different, a high proportion of both police officers and driving instructors reported that young drivers need training on merging and driving on slippery roads.

Many comparisons between police officers and driving instructors were not significant, indicating a strong level of agreement. Both groups similarly reported that young drivers require additional training in signaling, speed control, merging with traffic, driving on slippery roads, and identifying hazardous situations. Additionally, there was agreement that young drivers did not require additional training on using GPS or backup cameras, driving at dusk/dawn, driving in windy conditions, parallel parking, and parking on hills.

## 4. Discussion

The findings show that young drivers engage in a variety of unsafe driving behaviors, including driving while under the influence of alcohol and cannabis, consistent with previous studies [5,10,13,14,18,19,35,36]. Our data show that 6.2% and 18.1% reported using cannabis and alcohol prior to driving, respectively. These rates are consistent with other studies that examined young driver behaviors in large urban cities in Canada [1,3,5,17,18,19,20,22,35] and in other developed countries [9,10,11,12,13,15,16,36]. For example, other Canadian studies in large urban cities show that between 4.7% and 11.5% of young drivers were under the influence of alcohol and 2.3% and 16.3% were under the influence of cannabis while driving [5,19]. Our findings show that the prevalence of driving under the influence of alcohol is almost double that of cannabis, although prior studies show that young drivers in large urban locations (e.g., Toronto) perceive cannabis to be significantly safer compared to alcohol when driving [5]. This may explain why cannabis rates are nearly double that of alcohol in large urban cities [5,19] as it is perceived as a less mind-altering substance compared to alcohol [37]. Meanwhile, in smaller urban centers, young drivers may opt to use alcohol instead of cannabis as there is less stigma around its use [38].

More than one-fifth (22%) of our rural drivers consumed alcohol prior to driving, consistent with prior studies showing that young drivers living in rural areas have higher rates of alcohol-impaired driving compared to young drivers living in urban centers [39]. We also found that alcohol consumption in this study was significantly associated with eating food/drinking beverages, cellphone use, and speeding, with prior studies similarly reporting that drinking and driving were significantly associated with texting while driving [22] and speeding in young drivers living in Canadian urban centers [13]. Other studies have also found that alcohol consumption is associated with speeding and distracted driving, as well as tailgating, stunting, and difficulties maintaining lane position in young drivers [10,11,40].

Our sample also reported engaging in other dangerous driving behaviors such as speeding, cell phone use (texting), driving while fatigued, eating food and consuming beverages while driving, adjusting the radio, and driving with pets. These findings are consistent with prior research that shows high rates of cellphone use [17,25], driving while fatigued [12], and speeding [13,39]. One Canadian study found that 35.7% of young drivers (aged 16–20) used their cellphone while driving [17], a rate much lower than the present study (57.8%). This could be attributed to the differing judicial systems and the fines levied for cellphone use between Saskatchewan and other Canadian provinces where prior research took place [5,13,20]. Fines, for example, in Ontario are more expensive, costing between USD 615 and USD 3000 for the first offense [41], compared to USD 580 and four demerit points in Saskatchewan [42]. Alternatively, there is less traffic in smaller urban centers compared to large urban centers, and drivers may feel as though they can safely operate their vehicles while using their cellphones (e.g., less stimulation). Additionally, approximately 63% of young drivers reported driving while fatigued [12] and 33.7–78.6% of young drivers admit to speeding (i.e., exceeding the posted speed limit by more than 10 km per hour) [13,39], whereas our findings show that 76.2% of young drivers drive while fatigued and 40.9% often drive faster than the posted speed limit. Urban cities in Saskatchewan have either single or double-lane roadways, have slower speed limits (i.e., 90 km/hr in the city), and have less traffic, whereas roadways in bigger urban cities are multi-lane highways with heavy traffic and faster speed limits (i.e., 100 km/h). As a result, young drivers in Saskatchewan may engage in speeding (with the lower speed limits) and engage in more risk-taking behaviors, consistent with findings from prior studies [43,44]. Additionally, it is likely young drivers in Saskatchewan may also be more fatigued when driving given the monotony of the driving routes, in addition to the lack of stimuli on the roadways.

Our study found that the most challenging situations faced by young drivers are night driving, driving in poor weather and road conditions, and encountering/evading animals. While there is a lack of data on the specific driving situations that are most challenging, prior studies have reported reasons for young driver crashes, which highlights opportunities for tailoring training programs and/or messaging about safe driving. For example, crashes in young drivers are significantly associated with driving at night (i.e., between the hours of midnight and 6:00 AM) [44,45], poor weather and road conditions [6,46], and wildlife [47]. Meanwhile, police reported that distracted driving, including using a cell phone, was also the most common reason for crashes, followed by driving too fast for conditions and driver inexperience/confusion. These findings are supported by prior studies that show that crashes are caused by cellphone use [4,10,11,17], driving too fast for road conditions [48], and driver inexperience [12,17]. Comparatively, driving instructors reported the most challenging situations for young drivers were related to lane positioning, checking blind spots, identifying potentially dangerous situations, including driving in poor weather conditions (snow and slippery conditions), avoiding pedestrians, and speed control.

Many of these challenges (e.g., night driving, driving in bad weather conditions), as described by young drivers, police, and driving instructors, are taught in a limited fashion as part of licensing training programs. Consequently, young drivers may lack the necessary experience and training when driving in these situations, especially in complex or difficult conditions, which may increase the risk of a crash. Additionally, although all young drivers receive driver training prior to receiving their full license, the content between provincial licensing authorities differs in Canada. Saskatchewan is characterized by having small cities with a significant number of the population living in rural areas. While the driver training programs can enhance the experience of unique challenges SK drivers may face, such as driving on gravel roads, encountering animals, and driving in icy and slippery conditions, there are limited opportunities to practice in these environments, and in some cases, it is not practiced at all. To improve young driver skills and awareness of these challenging environments and conditions, SGI should consider revisions to their High School Driver Education program to include more in-class and in-car driving lessons. More specifically, additional training modules (videos) are needed on distracted driving (i.e., cellphone use) and the potential consequences, how to select appropriate speeds for different weather and road conditions, and how to identify potentially dangerous situations. Moreover, additional in-car driving lessons (beyond the 6 h currently required) are needed for young drivers to gain more experience with vehicle placement, maneuverability, and mirror checking. Alongside additional in-car driving lessons, the use of driving simulators may offer young drivers the opportunity to learn about these conditions and build confidence prior to being exposed to these situations on the road. Driving simulation can replicate the driving experience by presenting sensory (visual, auditory, kinematic, etc.) information to drivers and allowing them to practice specific driving skills in a controlled environment [49]. Additionally, one advantage of using driving simulators for training is the ability to control the type of road, weather, lighting conditions, traffic, pedestrians, and wildlife. Studies show that driving simulators are beneficial for training a wide range of driving skills and maneuvers in young drivers [50,51,52,53,54,55], including basic vehicle control (e.g., accelerating, braking, lane position, etc.) [52], hazard anticipation [51], attention [55], and visual search skills [54]. This versatility can create new possibilities for improving the driver training experience for young drivers and reducing the high crash risk observed in this group [2].

## 5. Recommendations

There are several policy or intervention opportunities to reduce the risk of young driver crashes in rural areas. Given the high rates of impaired driving, the development of ridesharing programs and better alternative transportation options is needed. In Saskatchewan, ridesharing programs such as Uber are restricted to major cities such as Regina and Saskatoon. Smaller cities that are just outside these larger cities do not have access to Uber. Additionally, there are limited taxi services in smaller communities and rural areas. Consequently, there is an inability to use ridesharing or taxi services to safely return home after consuming alcohol or drugs. A California study reported that ride-sharing platforms such as Uber have reduced traffic fatalities by 4% overall and reduced alcohol traffic fatalities by 6% [56].

Another possibility is the development of community programs. For example, the “Road Crew” program that serves six Wisconsin rural counties using volunteer drivers and taxis from the community provides rides for people who have consumed alcohol. Data shows that the primary users were males between the ages of 21 and 34, and after 97,000 trips, they prevented an estimated 140 alcohol-related crashes [57]. On an individual level, these programs have the potential of reducing accidents and saving lives. On a large scale, however, these programs are more difficult to implement and are often needed to observe significant program effects. Other possibilities mentioned in the literature are the inclusion of ignition interlocks in vehicles. In this respect, no driver who has consumed more than a pre-determined amount of alcohol (e.g., 0.04 blood alcohol concentration) would be allowed to drive [58,59]. However, should an individual not be able to drive, then alternative transportation must be available. Additionally, the ignition interlock cannot detect cannabis or other drugs, limiting its ability to identify impairments other than alcohol.

We also found that young drivers reported speeding, having difficulties driving at night, and maneuvering around animals. In Saskatchewan, there is a lack of lighting on rural highways and roads, increasing the risk of accidents. For example, the lack of lights increases the difficulty of staying within the respective lane and viewing animals on the road. The concentration needed to focus on the roadway may also increase cognitive load, leading to speeding. To address this issue, one option is to increase road visibility through overhead lights or lights on the roadway itself. Prior studies show that implementing rural highway lighting reduces nighttime injury crashes by 28% [60]. Other options include widening the edge lines to improve roadway visibility, placing rumble strips on the shoulder and center line, and implementing chevron markings on curves [60]. These measures show a 25% reduction in nighttime crashes and a 16% reduction in fatal and injurious crashes [60].

## 6. Limitations

This study has several limitations, including that the questionnaires completed by all respondents were based on self-report, which is subject to recall and social desirability bias. Additionally, we do not know the response rate and cannot determine if our sample is representative of the typical young driver, driving instructor, or police officer. However, we do know there are 219 Class 5 driving schools listed in Saskatchewan, meaning our response rate for driving instructors is approximately 28% (62/219). Additionally, we did not find any significant association between distracted driving behaviors and crashes. This is likely due to the small number of crashes reported in our sample. We also did not ask young drivers to provide a reason as to why they were involved in a crash or why they had received a citation. And lastly, we could not perform gender (male vs. female) or location (urban vs. rural) comparisons given the small cell sizes for some variables, including alcohol and drug use. Our sample of police officers was also from urban contexts, potentially biasing the findings to urban drivers; however, in Saskatchewan, it is important to note that many urban cities include rural municipalities within the city boundaries. Thus, it is likely that our sample of police officers were patrolling and enforcing laws in rural areas.

## 7. Conclusions

Young drivers living in smaller cities and rural communities have unique challenges, including interactions with wildlife, driving on gravel roads, and driving in poor weather and road conditions (e.g., ice, snow). We also found high rates of speeding and distracted driving behaviors (e.g., texting and driving). Opportunities for young drivers to be exposed to these scenarios during driver training, through tailored education and simulation, are critical for increasing awareness and for reducing crash risk. Additionally, we found that almost one-fifth of young drivers reported driving under the influence of alcohol, suggesting interventions are needed in rural areas (e.g., ridesharing; better public transportation, including taxis).

## Figures and Tables

**Table 1:** Self-Reported Alcohol Use and Driving Behaviors of Young Drivers.

	Consumed Alcohol Before Driving		Significance
	Yes (n = 35)	No (n = 158)	
**Apply makeup**			χ^2^ = 0.27, *p* = 0.443
Sometimes	2 (5.7%)	6 (3.8%)	
Never	33 (94.3%)	152 (96.2%)	
**Loud music**			χ^2^ = 4.25, *p* = 0.120
Often	22 (62.9%)	69 (43.7%)	
Sometimes	11 (31.4%)	74 (46.8%)	
Never	2 (5.7%)	15 (9.5%)	
**Wear headphones**			χ^2^ = 0.41, *p* = 0.816
Often	0 (0.0%)	1 (0.6%)	
Sometimes	1 (2.9%)	7 (4.4%)	
Never	34 (97.1%)	150 (94.9%)	
**Eat/Drink**			**χ^2^ = 6.89, *p* = 0.032**
Often	10 (28.6%)	23 (14.6%)	
Sometimes	25 (71.4%)	119 (75.3%)	
Never	0 (0.0%)	16 (10.1%)	
**Adjust GPS**			χ^2^ = 3.87, *p* = 0.276
Often	2 (5.7%)	15 (9.5%)	
Sometimes	17 (48.6%)	55 (34.8%)	
Never	1 (2.9%)	17 (10.8%)	
No GPS	15 (42.9%)	71 (44.9%)	
**Adjust Radio**			χ^2^ = 5.44, *p* = 0.142
Often	19 (54.3%)	63 (39.9%)	
Sometimes	16 (45.7%)	77 (48.7%)	
Never	0 (0.0%)	17 (10.8%)	
**Look at passengers**			χ^2^ = 3.09, *p* = 0.214
Often	0 (0.0%)	5 (3.2%)	
Sometimes	21 (60.0%)	72 (45.6%)	
Never	14 (40.0%)	81 (51.3%)	
**Drive with pets**			χ^2^ = 5.51, *p* = 0.063
Yes	15 (42.9%)	53 (33.5%)	
No	9 (25.7%)	74 (46.8%)	
N/A	11 (31.4%)	31 (19.6%)	
**Handle cellphone**			**χ^2^ = 11.7, *p* = 0.003**
Often	4 (11.4%)	8 (5.1%)	
Sometimes	25 (71.4%)	74 (46.8%)	
Never	6 (17.1%)	76 (48.1%)	
**Merging or changing lanes**			χ^2^ = 1.15, *p* = 0.564
Often	1 (2.9%)	4 (2.5%)	
Sometimes	15 (42.9%)	53 (33.5%)	
Never	19 (54.3%)	101 (63.9%)	
**Tailgating**			χ^2^ = 2.95, *p* = 0.229
Often	1 (2.9%)	4 (2.5%)	
Sometimes	12 (34.3%)	33 (20.9%)	
Never	22 (62.9%)	121 (76.6%)	
**Night driving**			χ^2^ = 2.86, *p* = 0.240
Often	0 (0.0%)	10 (6.3%)	
Sometimes	16 (45.7%)	58 (36.7%)	
Never	19 (54.3%)	90 (57.0%)	
**Passing**			χ^2^ = 0.22, *p* = 0.895
Often	0 (0.0%)	1 (0.6%)	
Sometimes	10 (28.6%)	45 (28.5%)	
Never	25 (71.4%)	112 (70.9%)	
**Gravel roads**			χ^2^ = 0.67, *p* = 0.714
Yes	9 (25.7%)	31 (19.6%)	
No	24 (68.6%)	116 (73.4%)	
N/A	2 (5.7%)	11 (7.0%)	
**Extreme weather**			χ^2^ = 0.17, *p* = 0.685
Yes	22 (62.9%)	105 (66.5%)	
No	13 (37.1%)	53 (33.5%)	
**Bright sun**			χ^2^ = 0.67, *p* = 0.716
Often	1 (2.9%)	9 (5.7%)	
Sometimes	13 (37.1%)	51 (32.3%)	
Never	21 (60.0%)	98 (62.0%)	
**Speeding**			**χ^2^ = 9.58, *p* = 0.008**
Often	22 (62.9%)	57 (36.1%)	
Sometimes	10 (28.6%)	90 (57.0%)	
Never	3 (8.6%)	11 (7.0%)	
**Wear seatbelt (driver)**			**χ^2^ = 4.54, *p* = 0.033**
Often	34 (97.1%)	158 (100.0%)	
Sometimes	1 (2.9%)	0 (0.0%)	
Never	0 (0.0%)	0 (0.0%)	
**Wear seatbelt (passenger)**			**χ^2^ = 4.40, *p* = 0.036**
Often	31 (88.6%)	153 (96.8%)	
Sometimes	4 (11.4%)	5 (3.2%)	
Never	0 (0.0%)	0 (0.0%)	
**Road rage**			χ^2^ = 0.16, *p* = 0.924
Often	2 (5.7%)	11 (7.0%)	
Sometimes	13 (37.1%)	62 (39.2%)	
Never	20 (57.1%)	85 (53.8%)	
**Drive fatigued**			χ^2^ = 3.62, *p* = 0.057
Yes	31 (88.6%)	116 (73.4%)	
No	4 (11.4%)	42 (26.6%)	
**Fell asleep driving**			χ^2^ = 0.51, *p* = 0.474
Yes	3 (9.7%)	7 (6.0%)	
No	28 (90.3%)	109 (94.0%)	

Bold indicates a significant relationship (i.e., *p*-value < 0.05).

**Table 2:** Common Self-Reported Distracted Driving Behaviors in Young Drivers (N = 193).

Distraction	Often	Sometimes	Never
Apply makeup	0 (0%)	8 (4.1%)	185 (95.9%)
Listen to loud music	91 (47.2%)	85 (44.0%)	17 (8.8%)
Wear headphones	1 (0.5%)	8 (4.1%)	184 (95.3%)
Eat or drink	33 (17.1%)	144 (74.6%)	16 (8.3%)
Adjust navigation systems *	17 (8.8%)	72 (37.3%)	18 (9.3%)
Adjust radio *	82 (42.5%)	93 (48.2%)	17 (8.8%)
Look at passengers to talk	5 (2.6%)	93 (48.2%)	95 (49.2%)
Buckle their pets *	15 (22.1%)	13 (19.1%)	40 (58.8%)
Use their cell phone	12 (6.2%)	99 (51.3%)	82 (42.5%)

Note: * 86 drivers (44.6%) did not have built-in navigation systems, 1 (0.50%) did not have a radio, and 42 (21.8%) did not have pets.

**Table 3:** Self-Reported Problematic Driving Situations for Young Drivers (N = 193).

Driving Situation	Often	Sometimes	Never
Merging/changing lanes	5 (2.6%)	68 (35.2%)	120 (62.2%)
Night driving	10 (5.2%)	74 (38.3%)	109 (56.5%)
Passing other vehicles	1 (0.5%)	55 (28.5%)	137 (71.0%)
Bright/sunny conditions	10 (5.2%)	64 (33.2%)	119 (61.7%)
Wear a seatbelt while driving	192 (99.5%)	1 (0.5%)	0 (0.0%)
Wear a seatbelt as passenger	184 (95.3%)	9 (4.7%)	0 (0.0%)

**Table 4:** Proportion of incidents that police give citations for to young drivers on different roadways (N = 65).

Citation	Intersections	Main Streets	Residential Streets	Side Roads	Highways
Speeding	5 (7.7%)	32 (49.2%)	14 (21.5%)	12 (18.5%)	46 (70.8%)
Impaired	8 (12.3%)	43 (66.2%)	18 (27.7%)	12 (18.5%)	25 (38.5%)
Distracted	39 (60.0%)	41 (63.1%)	15 (23.1%)	7 (10.8%)	11 (16.9%)
Failure to stop	54 (83.1%)	9 (13.8%)	18 (27.7%)	12 (18.5%)	11 (16.9%)
Improper signaling	25 (38.5%)	33 (50.8%)	16 (24.6%)	6 (9.2%)	17 (26.2%)

Note: Respondents could select more than one answer.

**Table 5:** Areas of agreement between police officers and driving instructors regarding additional training.

Need Training	Police Officers (n = 65)	Driving Instructors (n = 62)	χ^2^ Significance
Using GPS			
No	63 (97%)	60 (97%)	χ^2^ = 0.002, *p* = 0.96
Yes	2 (3%)	2 (3%)	
Using Backup Cameras			
No	61 (94%)	60 (97%)	χ^2^ = 0.60, *p* = 0.44
Yes	4 (6%)	2 (3%)	
Using cruise control			
No	53 (82%)	60 (97%)	**χ^2^ = 7.51, *p* = 0.006**
Yes	12 (18%)	2 (3%)	
Signaling			
No	36 (55%)	43 (69%)	χ^2^ = 2.63, *p* = 0.11
Yes	29 (45%)	19 (31%)	
Shifting gears			
No	64 (99%)	55 (89%)	**χ^2^ = 5.11, *p* = 0.024**
Yes	1 (1%)	7 (11%)	
Speed control			
No	29 (45%)	24 (39%)	χ^2^ = 0.46, *p* = 0.50
Yes	36 (55%)	38 (61%)	
Pedal use			
No	62 (95%)	52 (84%)	**χ^2^ = 4.58, *p* = 0.032**
Yes	3 (5%)	10 (16%)	
Lane positioning			
No	45 (69%)	17 (27%)	**χ^2^ = 22.2, *p* < 0.001**
Yes	20 (31%)	45 (73%)	
Merging with traffic			
No	31 (48%)	24 (39%)	χ^2^ = 1.04, *p* = 0.31
Yes	34 (52%)	38 (61%)	
Checking blind spots			
No	33 (51%)	19 (31%)	**χ^2^ = 5.32, *p* = 0.021**
Yes	32 (49%)	43 (69%)	
Dawn/Dusk driving			
No	53 (82%)	56 (90%)	χ^2^ = 2.01, *p* = 0.16
Yes	12 (18%)	6 (10%)	
Night-time driving			
No	47 (72%)	56 (90%)	**χ^2^ = 6.72, *p* = 0.010**
Yes	18 (28%)	6 (10%)	
Driving in rain			
No	38 (59%)	49 (79%)	**χ^2^ = 6.22, *p* = 0.013**
Yes	27 (41%)	13 (21%)	
Driving in snow			
No	15 (23%)	32 (52%)	**χ^2^ = 11.1, *p* < 0.001**
Yes	50 (77%)	30 (48%)	
Windy conditions			
No	53 (82%)	46 (74%)	χ^2^ = 1.00, *p* = 0.32
Yes	12 (18%)	16 (26%)	
Slippery roads			
No	20 (31%)	25 (40%)	χ^2^ = 1.27, *p* = 0.26
Yes	45 (69%)	37 (60%)	
Navigating heavy traffic			
No	39 (60%)	24 (39%)	**χ^2^ = 5.75, *p* = 0.016**
Yes	26 (40%)	38 (61%)	
Traffic Signs/Signals			
No	49 (75%)	26 (42%)	**χ^2^ = 14.7, *p* < 0.001**
Yes	16 (25%)	36 (58%)	
Navigating Pedestrians			
No	52 (80%)	31 (50%)	**χ^2^ = 12.6, *p* < 0.001**
Yes	13 (20%)	31 (50%)	
Navigating Cyclists			
No	56 (86%)	38 (61%)	**χ^2^ = 10.2, *p* = 0.001**
Yes	9 (14%)	24 (39%)	
Identifying hazardous situations			
No	34 (52%)	24 (39%)	χ^2^ = 2.37, *p* = 0.12
Yes	31 (48%)	38 (61%)	
Parallel parking			
No	51 (79%)	46 (74%)	χ^2^ = 0.32, *p* = 0.57
Yes	14 (21%)	16 (26%)	
Parking on hills			
No	62 (95%)	58 (94%)	χ^2^ = 0.21, *p* = 0.65
Yes	3 (5%)	4 (6%)	

Note: Bold = statistical significance (i.e., *p*-value < 0.05).

## Data Availability

The original contributions presented in this study are included in the article. Further inquiries can be directed to the corresponding author(s).
